# Isolation of human cutaneous immune cells for single-cell RNA sequencing

**DOI:** 10.1016/j.xpro.2023.102239

**Published:** 2023-04-28

**Authors:** Ashley A. Hailer, David Wu, Abdullah El Kurdi, Michelle Yuan, Raymond J. Cho, Jeffrey B. Cheng

**Affiliations:** 1 Department of Dermatology, University of California, San Francisco, San Francisco, CA 94107, USA; 2 Dermatology, Veterans Affairs Medical Center, San Francisco, CA 94121, USA; 3 Department of Biochemistry and Molecular Genetics, American University of Beirut, Beirut, Lebanon

**Keywords:** Cell Isolation, Flow Cytometry/Mass Cytometry, Immunology, Molecular Biology, RNAseq, Single Cell

## Abstract

Single-cell RNA sequencing (scRNA-seq) allows for high-resolution analysis of transcriptionally dysregulated cell subpopulations in inflammatory diseases. However, it can be challenging to properly isolate viable immune cells from human skin for scRNA-seq due to its barrier properties. Here, we present a protocol to isolate high-viability human cutaneous immune cells. We describe steps for obtaining and enzymatically dissociating a skin biopsy specimen and isolating immune cells using flow cytometry. We then provide an overview of downstream computational techniques to analyze sequencing data.

For complete details on the use and execution of this protocol, please refer to Cook et al. (2022)^1^ and Liu et al. (2022).^2^

## Highlights

• Isolation of viable immune cells from human skin for single-cell RNA sequencing
• Manual dissociation/enzymatic digestion without proprietary reagents
• Basic workflow for computational analysis of immune cell subpopulations

## Abstract (editor highlights)

Publisher’s note: Undertaking any experimental protocol requires adherence to local institutional guidelines for laboratory safety and ethics.

## Graphical abstract

**Figure:**
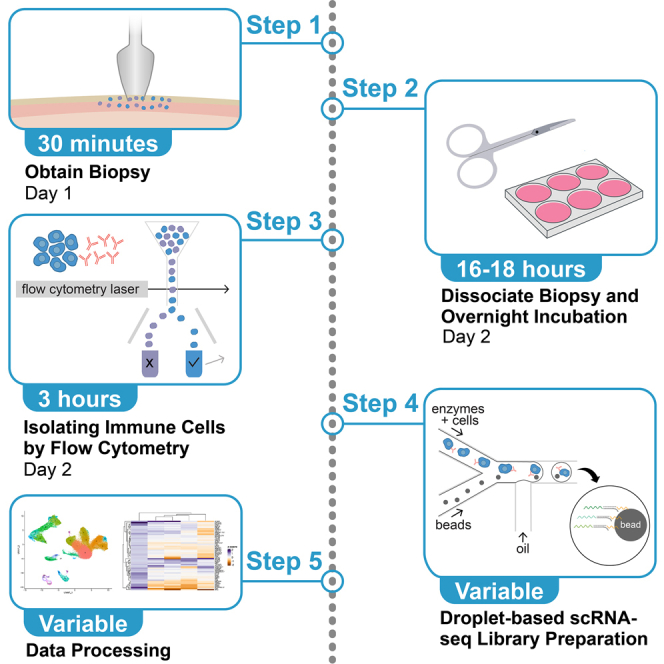

## Before you begin

This protocol describes the isolation of a high viability single-cell suspension of cutaneous immune cells from a 6 mm full-thickness punch skin biopsy for single-cell RNA-sequencing. This isolation occurs over two days. The initial day entails biopsy collection and manual sample dissociation, followed by overnight enzymatic digestion. The second consecutive day isolates a single-cell suspension of CD45^+^ immune cells for sequencing. We utilize this protocol for inflammatory skin disease (e.g., psoriasis vulgaris and atopic dermatitis) and healthy skin samples. We also further describe a basic computational workflow for processing the scRNA-seq data outputs.

Before obtaining the biopsy, ensure that all downstream reagents are prepared and that there is access to a FACS sorter on the second day. The protocol must be completed efficiently without stopping points to decrease cellular stress for optimal results.

We typically utilize at least a 6 mm full-thickness skin punch biopsy. After the biopsy procedure, the skin sample should be stored in cell media consisting of RPMI-1640 media supplemented with 10% Fetal Bovine Serum (FBS), 100 IU/mL Penicillin, 100 μg/mL Streptomycin, and 10 mM HEPES at 4°C until the isolation procedure begins, which typically occurs in the late afternoon prior to the 16–18 h overnight digestion. Depending on when the biopsy is taken, the biopsy will remain at 4°C for up to several hours. All downstream isolation steps should be conducted in a biosafety cabinet. On the second day of the protocol, all reagents and centrifuges should be kept at 4°C.

### Institutional permissions

Written informed consent for human skin biopsies should be under protocols approved by the institution’s Institutional Review Board (IRB) that allow for genomic data sharing. This study was conducted using protocols approved by the University of California, San Francisco’s Human Research Protection Program Institutional Review Board.

## Key resources table

**Table:** 

REAGENT or RESOURCE	SOURCE	IDENTIFIER
**Antibodies**		

DAPI (4′,6-diamidino-2-phenylindole)	Sigma-Aldrich	D9542-1MG
CD45 Mouse anti-Human, APC, Clone: HI30, eBioscience (approximately a 1:30 dilution)	Thermo Fisher Scientific	5014985

**Critical commercial assays**		

Chromium Next GEM Single Cell 3ʹ Reagent Kit v3.1	10× Genomics	

**Deposited data**		

scRNA-seq BAM files	Cook et al.^1^	European Genome-Phenome Archive (EGA) accession number EGA: S00001006716
scRNA-seq BAM files	Liu et al.^2^	European Genome-Phenome Archive (EGA) accession number EGA: S00001005271

**Software and algorithms**		

R version 4.0.5	R Foundation	https://www.r-project.org
Cell Ranger version	10× Genomics	https://support.10xgenomics.com/single-cell-gene-expression/software/pipelines/latest/installation
Seurat 4.0.2	Stuart et al.^3^	https://github.com/satijalab/seurat
Harmony 0.1.0	Korsunsky et al.^4^	https://github.com/immunogenomics/harmony
ScDblFinder 1.12.0	Germain et al.^5^	https://github.com/plger/scDblFinder
MAST 1.24.0	McDavid et al.^6^	https://github.com/RGLab/MAST/

**Other**		

Fetal bovine serum (heat inactivated) (FBS)	UCSF Cell Culture Facility	CCFAQ008
Phosphate buffered saline without calcium or magnesium (PBS)	UCSF Cell Culture Facility	CCFAE001
DNase I	Sigma-Aldrich	DN25
Collagenase IV	Worthington Biochemical Corp.	LS004188
Penicillin/Streptomycin	Thermo Fisher Scientific	15140122
1 M HEPES pH 7.4	UCSF Cell Culture Facility	CCFGL001
Cell staining buffer	BioLegend	420201
RPMI-1640 with 2.0 g/L NaHCO_3_	UCSF Cell Culture Facility	CCFAE001
Hardened fine scissors	Fine Science Tools	14090-11
Corning Falcon 100 μm cell strainer	Fisher Scientific	352360
Wide-orifice, low-retention filter LTS tips	Mettler-Toledo Rainin	30389241
Corning™ 5 mL Falcon™ round-bottom polystyrene test tubes with cell strainer snap cap	Fisher Scientific	352235
Corning™ 15 mL polypropylene centrifuge tubes, sterile	Fisher Scientific	430052
Microcentrifuge tubes, 0.8 mL graduated, flat cap tube, low retention	Fisher Scientific	02-681-311

## Materials and equipment

**Table:** Cell Culture Media

Reagent	Final concentration	Amount per biopsy
RPMI 1640	N/A	3.52 mL
Fetal Bovine Serum (Heat Inactivated)	10%	400 μL
HEPES (1 M)	10 mM	40 μL
Penicillin and Streptomycin	Penicillin 100 IU/mL, Streptomycin 100 μg/mL	40 μL of 100× solution
**Total**	**N/A**	**4 mL**

Penicillin and Streptomycin are in a 100× solution and stored at −20°C. Store cell culture media at 4°C. Dispose of cell culture media after one month.

**Table:** Digestion Buffer

Reagent	Final concentration	Amount per biopsy
Cell Culture Media	N/A	3.56 mL
Collagenase IV	200 U/mL	400 μL of 10× solution
DNase I	0.1 mg/mL	40 μL
**Total**	**N/A**	**4 mL**

We reconstitute Collagenase IV in RPMI-1640 in a 10× concentration of 2,000 U/mL. For Collagenase IV, we make 10 mL, aliquot 1 mL in microcentrifuge tubes, and have stored at −20°C for up to 3 months. The manufacturer does not make reconstituted storage claims, however, they note that multiple laboratories use stocks for 2–3 months. We reconstitute DNase I at 10 mg/mL in sterile water. The manufacturer notes that DNase I at this concentration will retain at least 90% of activity when stored at −20°C for up to one year. Avoid freeze/thawing. Make a fresh digestion buffer for each biopsy.

**Table:** Wash Buffer

Reagent	Final concentration	Amount
RPMI 1640	90%	40 mL
Fetal Bovine Serum (Heat Inactivated)	10%	10 mL
**Total**	**N/A**	**50 mL**

Store at 4°C. During the protocol, keep at 4°C or on ice. Dispose of after one month.

**Table:** FACS Buffer

Reagent	Final concentration	Amount
Phosphate Buffered Saline without Calcium or Magnesium (PBS)	98%	48 mL
Fetal Bovine Serum (Heat Inactivated)	2%	2 mL
**Total**	**N/A**	**50 mL**

Store at 4°C. During the protocol, keep at 4°C or on ice. Dispose of after one month.

**Table:** Cytokine Cocktail for CD45-APC Staining

Reagent	Final concentration	Amount
Cell Staining Buffer	97%	97 μL
CD45 Mouse anti-Human, APC, Clone: HI30, eBioscience	3%	3 μL
**Total**	**N/A**	**100 μL**

Make fresh for each skin prep on the day of FACS staining. Store at 4°C protected from light until use.

## Step-by-step method details

### Day one: Obtaining the biopsy

**Timing: 30 min–1 h**

In this step, the skin biopsy is obtained for downstream processing.

1. A 6 mm skin punch biopsy is collected in a sterile 15 mL conical tube with 10 mL of RPMI-1640, supplemented with 10% Fetal Bovine Serum (FBS), 100 IU/mL Penicillin, 100 μg/mL Streptomycin, and 10 mM HEPES and kept on ice during transport.

### Day one: Manual dissociation of sample by mincing

**Timing: 30 min**

In this step, you will begin the enzymatic dissociation and incubate the cells overnight for 16–18 h.

***Note:*** The cells require overnight enzymatic digestion for 16–18 h, so you should begin the “Day One: Manual Mincing Dissociation of Sample” 16–18 h before you plan to start the protocol on the second day.

***Note:*** These steps are conducted at room temperature under sterile conditions in a biosafety hood. All cell work should use wide-bore pipette tips to decrease cell shearing.

2. Transfer the biopsy to a sterile cell culture dish to remove all subcutaneous fat and hair with scissors (Figure 1).

**Figure 1:**
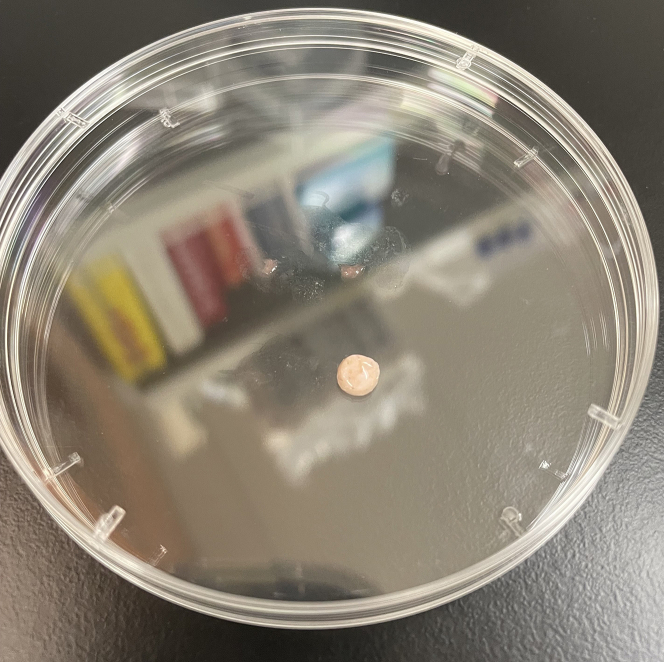
Skin biopsy specimen in sterile cell dish after removing subcutaneous fat and hair
3. Add 2 mL of the enzymatic digestion buffer to a 6-well plate.
4. Transfer the biopsy to the well containing 2 mL of digestion buffer.
5. Mince the tissue with scissors for 4 min (see Methods video S1 for an example of mincing and Figure 2 for the desired final consistency).

**Figure 2:**
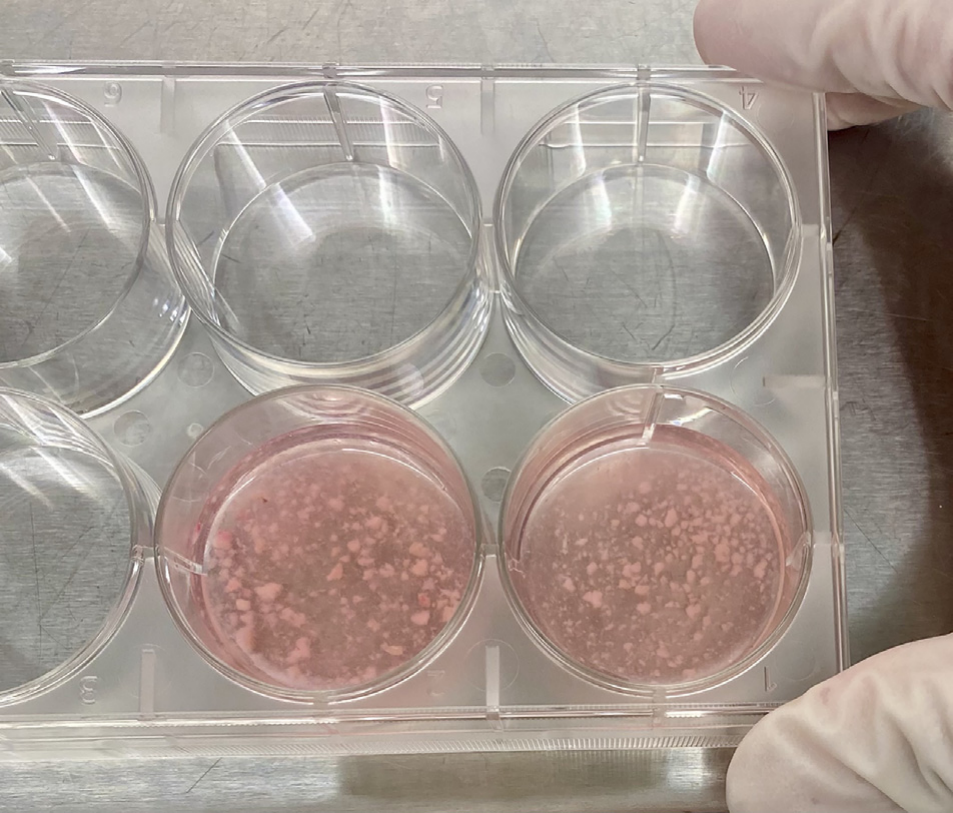
Appropriate skin specimen consistency after 4 min of manual mincing with scissors
6. Add 2 mL of the digestion buffer into the same well to bring to a total of 4 mL.
7. Incubate at 37°C with 5% CO_2_ overnight for a minimum of 16 h and a maximum of 18 h.

***Note:*** Cell yield will vary between samples and may depend upon the quality of mincing.

**CRITICAL:** Minimize the time the sample is not in media to maintain cell viability.

Methods video S1. Skin sample manual mincing with scissors technique, related to step 5

### Day two: Obtaining a single-cell suspension

**Timing: 15 min**

In this step, you will conclude the enzymatic dissociation and obtain a single-cell suspension to stain for CD45^+^ immune cells for subsequent flow cytometry sorting.

***Note:*** All buffers, centrifuges, and cells should be kept on ice or at 4°C to maintain cell viability, and cells should be processed efficiently. All centrifuge steps should be performed with a swinging bucket rotor.

8. In the 6-well plate containing the digested biopsy, gently pipette up and down 6–8 times to mix with a 5 mL serological pipette and transfer to a 50 mL conical tube (Methods video S2).
9. Rinse the same well with 4 mL of cold wash buffer and transfer to the same 50 mL conical tube.
10. Vigorously shake the tube for 30 s. Do not vortex as this may lyse or damage the cells.
11. Pre-wet a 100 μm filter with 1 mL wash buffer over a new 50 mL conical tube.
12. Pour the cell solution through the filter into the new 50 mL tube.
13. Rinse the original conical tube with 5 mL of wash buffer and pour over the filter to wash the filter. Allow the liquid to filter through thoroughly before removing and discarding the strainer.
14. Centrifuge the new conical tube containing the single cell suspension at 400 g for 5 min at 4°C.
15. Decant by pouring supernatant out. There will be approximately 100–200 μL of volume remaining. See Methods video S3 for further clarification.

**Figure 3:**
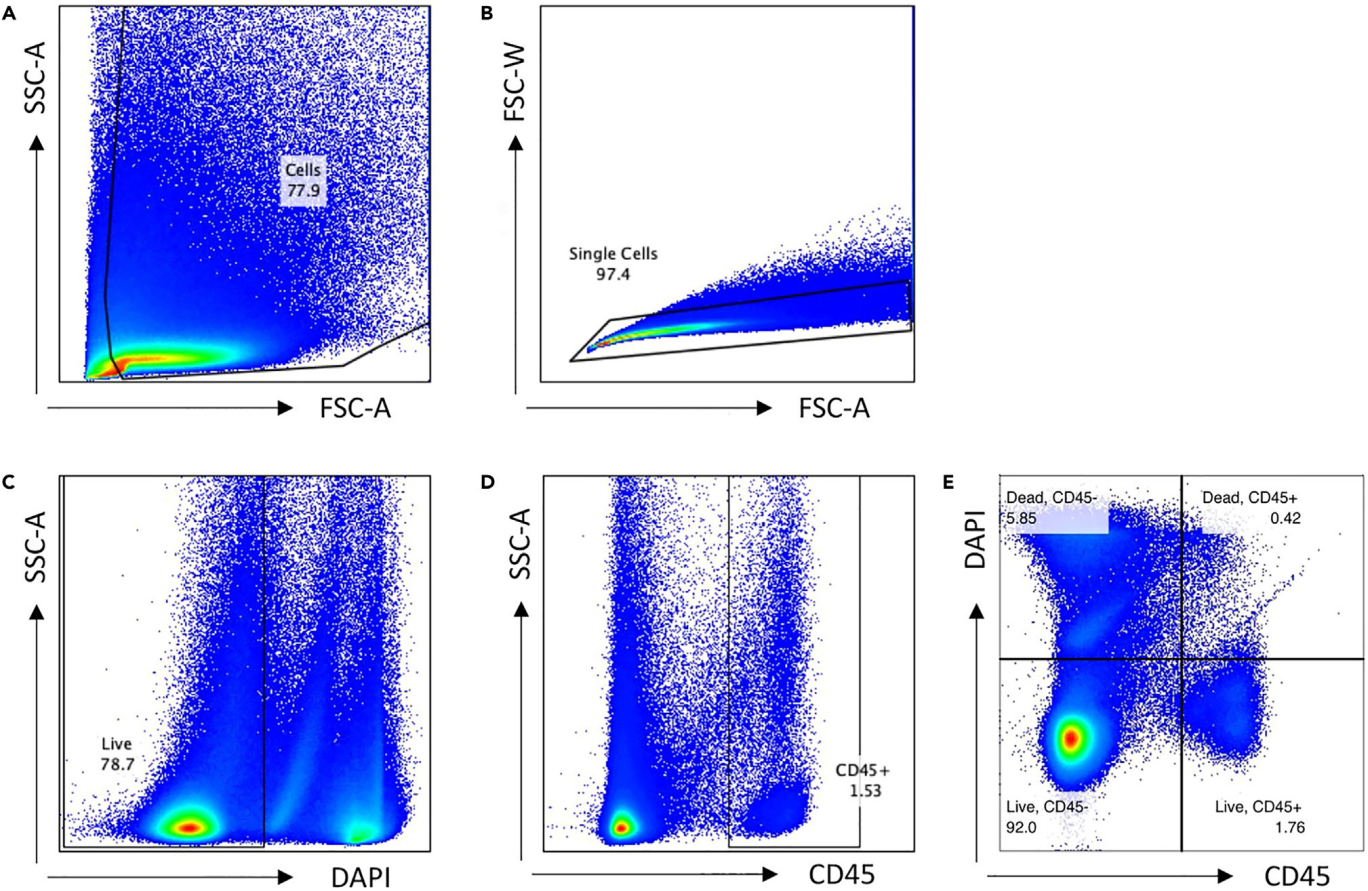
Flow cytometry gating methods (A) Excluding Debris with a Forward Scatter (FSC) versus Side Scatter (SSC) plot. (B) Excluding Doublets with a FSC-Area (A) versus FSC-Width (W) plot. (C) Exclusion of Dead Cells with a DAPI-A versus SSC-A plot. (D) Obtaining Live, Single, CD45^+^ Cells with a SSC-A versus CD45^+^ graph. (E) Optional method to plot DAPI Live Dead versus CD45 APC.

***Note:*** There is less cell loss with decanting relative to supernatant aspiration.

***Note:*** We do not utilize a red blood cell lysis step as CD45^+^ cells will be selected for in FACS sorting.

***Note:*** It is not necessary to conduct a debris removal step because the cells will be filtered in steps 12 and 28, undergo several wash steps, and debris will be gated out in the first plot of the FACS sorting step (See Figure 3A).

16. Resuspend in the remaining volume and transfer to a 96-well V-bottom plate.
17. Count the cells using a hemocytometer.
  a. Obtain a 7.5 μL aliquot of the cell suspension and add an equal amount of 0.4% Trypan Blue Solution.
  b. Gently mix, incubate at room temperature for 5 min.
  c. Transfer 10 μL to a hemocytometer chamber.

***Note:*** Typical yields will vary based on disease and diseased state. Ranges that we note for healthy, non-diseased skin are 300,000–600,000 cells and for inflamed skin ranges from 500,000 to 5 million cells.

Methods video S2. Day Two Pipetting to Mix the Sample, related to step 8

Methods video S3. Decanting Supernatant when in 50 mL or FACS tubes, related to step 15

### Day two: Staining the single-cell suspension for CD45^+^ immune cell sorting

**Timing: 45 min**

In this step, the immune cells will be marked for separation from the rest of the cell suspension with fluorochrome-labeled antibodies. The flow cytometer will achieve this isolation through scattered light and fluorescence-based sorting. These CD45^+^ immune cells will be the cells utilized for single-cell RNA-sequencing.

18. Centrifuge the 96-well plate at 400 g for 5 min at 4°C.
19. Discard the supernatant by tipping over the plate.
20. Resuspend the cells with 100 μL of antibody cocktail. The antibody cocktail will contain 97 μL of Cell Staining Buffer and 3 μL of CD45 Mouse Anti-Human APC (approximately a 1:30 dilution).

***Note:*** Researchers should conduct antibody titrations on optimization/test skin samples to ensure optimal antibody staining and ultimately optimal immune cell isolation.

***Note:*** When staining for flow cytometry, it is common practice to make an antibody cocktail as a master solution containing the conjugated antibodies and the staining buffer and resuspend the dry pellet with 100 μL of this solution. This is especially beneficial when there are many samples or multiple antibodies to stain for. By creating an antibody cocktail, you increase the volumes pipetted, create a uniform solution, and can quickly and efficiently stain numerous samples at a time. Always make enough volume for the number of samples (plus 1–2) for the antibody cocktail.

***Note:*** Other fluorophores may be used. However, we have noted that APC allows for a bright signal and minimal spectral overlap with DAPI. Please ensure that the selection of fluorophores is compatible with your cytometer. Per the manufacturer, the CD45 Mouse Anti-Human antibody that we utilize has an Excitation Range of 633–647 nm, an Emission Spectrum of 660 nm, and is excited by the Red Laser (637 nm) with a BandPass Filter of 670/30 and LongPass Mirror of 650LP. DAPI can be excited by an ultraviolet (UV) or violet laser. We utilize the violet laser at 402 nm with a BandPass Filter of 431/28 and LongPass Mirror of 410LP. DAPI excites at 358 nm and has an emission maximum of 460 nm.

**CRITICAL:** Do not add DAPI at this point in the protocol. DAPI will be added at the end of the procedure, in step 38, just before sorting the sample in the FACS sorter.

21. Incubate for 30 min at 4°C in the dark.

**CRITICAL:** Flow cytometry antibodies are light-sensitive and can be photobleached. It is critical that the cells are incubated in the dark and protected from excess light downstream.

22. After incubation, add 150 μL of FACS buffer and pipette up and down three times to mix.

**CRITICAL:** To maximize viability, it is essential to be gentle with resuspending the cells.

23. Centrifuge the plate at 400 g for 5 min at 4°C.
24. Discard the supernatant by tipping over and resuspend with 200 μL of FACS buffer. Pipette up and down to resuspend three times.
25. Centrifuge the plate at 400 g for 5 min at 4°C.
26. Discard the supernatant by tipping over.
27. Resuspend cells in 200 μL FACS buffer.
28. Using the same pipette tip, transfer to a filtered FACS tube to filter the cells before sorting.
29. Wash the original well with 200 μL of FACS buffer and pass through the same filter to wash.
30. Fill a new 5 mL FACS tube with 3 mL of Cell Staining Buffer to collect the sorted cells.

***Note:*** When sorting cells, the concentration affects the sorting efficiency. We find that 400 μL is an appropriate volume for efficient sorting while minimizing sort time. With inflamed samples, we often have 1–2 million cells at this step, however, we have noted that 400 μL is appropriate with our highly inflamed samples that have had up to 5 million cells. An additional method to monitor this is with sort efficiency. If you have a higher number of cells (e.g., a highly inflamed skin sample), you may need to increase the final volume. While sorting, your cytometer may include a sort efficiency metric, which can aid in determining the best concentration for your sample. During the sort, if your sort efficiency falls below 70%, it is discarding 30% of your sorted, desired cells. This indicates that your sample may be too concentrated, and you should increase the buffer volume to decrease sorted cell loss.

### Cell sorting/gating preparation

**Timing: 30 min–1 h**

In this section, you will prepare your flow cytometry gating workflow as well as test your DAPI concentration. This should be conducted on optimization/test skin samples prior to obtaining patient biopsies that you intend to perform scRNA-seq on. If you have completed this workflow and created a gating template, you can proceed directly to the next section on Cell Sorting for CD45+ Cells with Flow Cytometry, skipping steps 31–35 and starting at step 36.

31. If not already prepared, set a sorting gate to capture cells on the Forward Scatter (FSC) versus Side Scatter (SSC) plot (Figure 3A).
32. If not prepared, take the gated population from the previous step and create a FSC-Area versus FSC-Width plot to exclude any doublets (Figure 3B).

***Note:*** You may additionally utilize a SSC-A versus SSC-H gate to exclude any remaining doublets.

33. If not prepared, plot the singlets on a DAPI-A versus SSC-A plot and create a gate to exclude any DAPI-positive cells (Figure 3C).

**CRITICAL:** The live cells will be DAPI-negative.

***Note:*** Our DAPI stock concentration is 1 μg/mL. We add DAPI for a final concentration of 1 μL per 1 million cells. However, it is beneficial to test this to ensure this concentration will allow for high viability.

34. If not prepared, plot the live, single cells on a SSC-A versus CD45^+^ APC plot and create a sorting gate for CD45^+^ cells (Figure 3D).

***Note:*** Alternatively, researchers may plot DAPI versus APC on a singular plot (see Figure 3E).

35. Create a sort layout to sort the live, single, CD45^+^ cells.

### Sorting for CD45^+^ cells with flow cytometry

**Timing: 30 min–1 h**

This step isolates the less abundant immune cell subpopulation and ensures high cell viability for sequencing by eliminating poor-quality cells and debris. These poor-quality cells include non-single cells and dead cells. We utilize the BD FACSAria Fusion Cell Sorter (BD Biosciences, Vienna, Austria). During this step, we collect all sorted CD45^+^ cells, as there will be cell loss in later wash steps.

***Note:*** The BD FACSAria Fusion Cell Sorter will sort 400 μL of our cell suspension in approximately 25 min. However, other cytometers may result in different time requirements for sorting. We have only conducted the sorting of up to three samples sequentially during the same day. The total sorting time was approximately 1.5 h and we have not seen any diminishment in cell quality at this number due to the extended preparation time due to processing multiple samples.

**CRITICAL:** Before obtaining patient biopsies, please ensure familiarity with flow cytometry and conduct optimization experiments to set PMT voltages, a gating template, and compensation controls. As referenced in steps 31–35, a gating template based on optimization samples will allow for timely and accurate flow sorting. This template will be created based on the single-stained samples to accurately set gating parameters.

**CRITICAL:** Ensure the cytometer has been turned on and calibrated before you bring your cells to sort. Prolonging the protocol by setting up the cytometer when the cells are ready to sort may decrease cell viability and increase cellular stress.

36. Turn the sorting pressure on to the lowest setting.
37. Open the previously created gating template (from the section: Cell sorting/gating preparation).
38. Add DAPI and flick mix the sample. Allow the DAPI to incubate for 5 min.

***Note:*** Our DAPI stock concentration is 1 μg/mL. We add DAPI for a final concentration of 1 μL per 1 million cells. However, it is beneficial to test this to ensure this concentration will allow for high viability.

39. Begin the sort with the pressure on the lowest setting, and sort into the previously prepared collection tube with 3 mL of Cell Staining Buffer.

**Figure 4:**
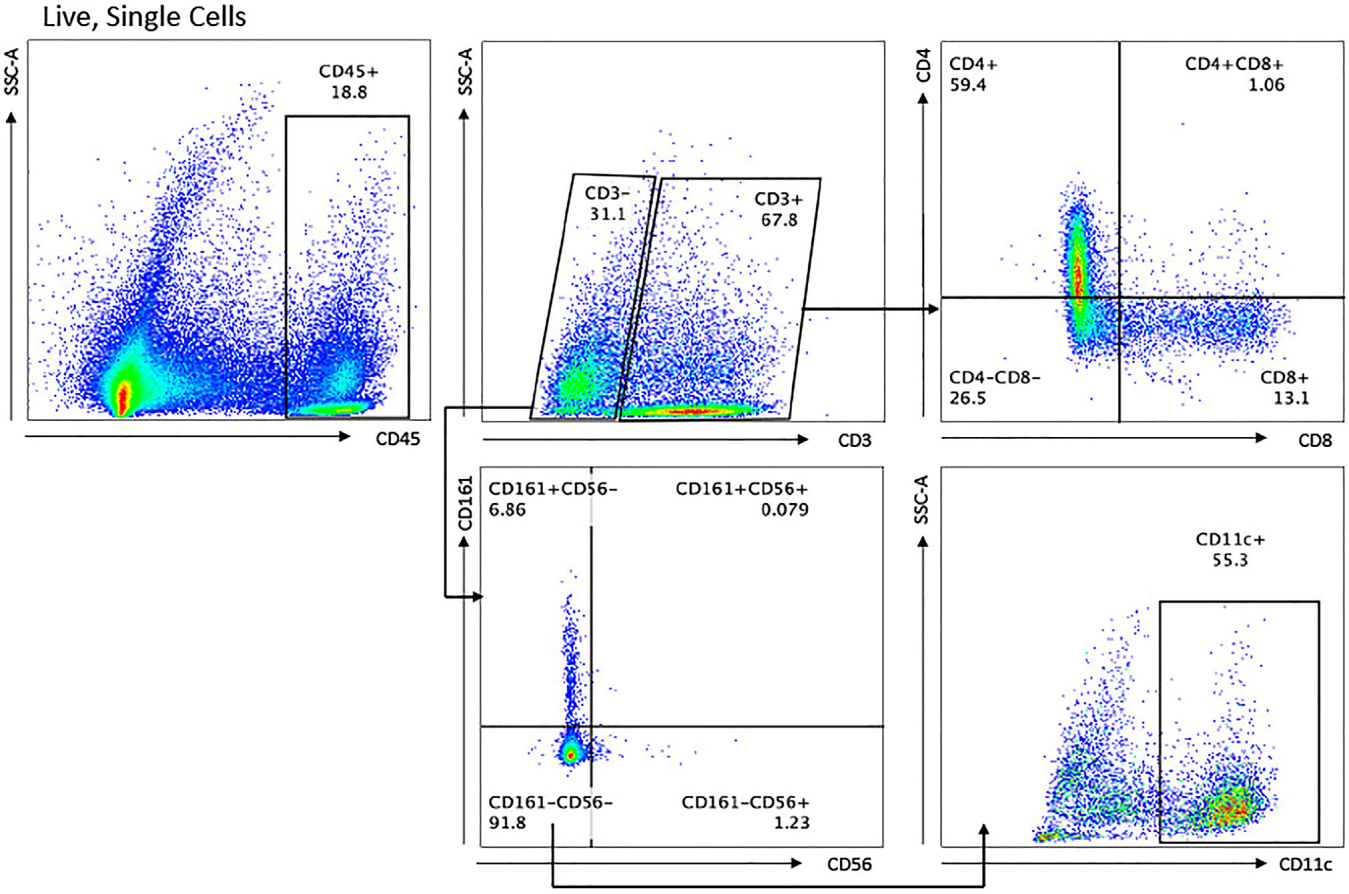
Immune cell subsets within the sorted CD45^+^ cell population

**CRITICAL:** Sorting should be conducted under the lowest pressure and flow setting to decrease cell stress.

***Note:***Figure 4 portrays selected expected immune cell populations, including T cells, natural killer cells, and dendritic cells.

### Day two: Preparing for 10× genomics chromium library preparation

**Timing: 30 min**

This step will ensure your CD45^+^ cells are at the proper volume and concentration for 10× Genomics Chromium Library Preparation.

***Note:*** All the below steps are conducted with the new FACS tube that contains your sorted CD45^+^ cells.

40. Add FACS buffer into the sorting tube to bring to a total of 5 mL.
41. Resuspend to wash the sides of the FACS tube with FACS buffer.
42. Centrifuge the tube at 400 g for 5 min at 4°C.
43. Decant by pouring off.
44. Centrifuge at 400 g for 1 additional minute at 4°C.
45. Resuspend in the remaining volume and transfer to a 96-well v-bottom plate.
46. Spin the plate at 400 g for 5 min at 4°C.
47. Decant the supernatant by tipping over.
48. Add 10 μL Cell Staining Buffer to a total volume of approximately 41 μL.

***Note:*** As noted in step 51, the final concentration will ideally be 700–1,200 cells/μL. By resuspending at 41 μL, after a volume loss of 7.5 μL for the cell count in step 50, the cell volume will likely be at the maximum volume that can be loaded on the Chromium chip for step 52.

49. Resuspend the cells and transfer to a Low Retention 0.8 mL Microcentrifuge Tube.
50. Perform a final cell count with a hemocytometer by taking a 7.5 μL aliquot of the cells and combining with 7.5 μL of Trypan Blue Solution, 0.4%. Gently mix, incubate at room temperature for 5 min, and then transfer 10 μL to a hemocytometer chamber.

***Note:*** The cell yield will vary between disease states. However, generally, healthy skin ranges from ∼5,000–20,000 cells at this step, and inflamed skin samples have an average of approximately 30,000 cells with a range of 5,000–50,000 cells depending on disease state.

51. Ideally, the final concentration of cells is 700-1,200 cells/μL.

***Note:*** If your concentration is above this, adjust to this concentration with Cell Staining Buffer and perform an additional cell count.

52. Proceed to desired single-cell RNA-sequencing library construction protocol (e.g., 10× Genomics Chromium Single Cell 3ʹ expression library kit). For scRNA-seq, please see the 10× Genomics latest recommendations for sequencing depth and parameters, but we typically utilize 20,000 read pairs/cell for Single Cell 3′ v3.1 libraries and Single Cell 5′ v2 libraries.

***Note:*** Minimize post-sorting time prior to utilizing cells for library preparation.

***Note:*** Researchers may desire an additional method to ensure cell viability prior to sequencing. As you will sort for live cells and conduct a live/dead cell count at the end of the protocol, researchers may identify additional tests for ensuring cell viability, but we have not found them to be necessary, as our scRNA-seq quality control metrics show a high percentage of fraction of reads in cells (representative of high cell viability).

***Note:*** To quantify and assess quality of our scRNA-seq libraries, we use the High Sensitivity DNA assay on the Agilent 2100 Bioanalyzer and either KAPA Library Quantification kit or a Qubit fluorometer.

## Expected outcomes

We utilize skin samples from inflammatory skin disease and healthy controls with this optimized protocol, obtaining an expected yield of ∼1,500–50,000 cells (CD45^+^ cells represent ∼1%–20% of live, single cells). When working with highly inflamed skin samples, CD45^+^ cell numbers are often increased, but there will be variable final cell numbers dependent on skin health status and individual patient variation. Ideally, this protocol should be optimized on more routine surgical skin discard samples before working with critical clinical samples intended for downstream analysis.

There are alternative protocols to isolate CD45^+^ cells from skin biopsies, e.g., the Miltenyi Biotec Whole Skin Dissociation Kit. However, we present here a relatively inexpensive, accessible, easily performed protocol to consistently obtain high-viability CD45^+^ cells from human skin without the need for proprietary reagents and equipment (e.g., gentleMACS™ Octo Dissociator (Miltenyi Biotec)). Other alternatives to flow sorting for immune cells include magnetic beads (e.g., EasySep™ Release Human CD45 Positive Selection Kit). However, these kits recommend starting with 100 million cells/mL, with a high starting viability. There will not be enough cells from a 6 mm skin punch biopsy to meet the minimum volumes required, even for the EasyPlate variation of this protocol, as 5 million cells in 5 μL is the minimum required volume. Also, the protocol requires high viability cells in this initial sort, which requires flow sorting in our protocol so an additional live/dead bead kit will be needed.

We have used this protocol for cells to utilize for 10× Genomics Chromium Single Cell 3ʹ expression library preparation,^1^^,^^2^ as well as for Chromium Single Cell 5′ Kits (v2 Chemistry Dual Index), combined with V(D)J enrichment allowing assessment of T or B cell V(D)J recombination. For the 10× Genomics Chromium Single Cell 3ʹ expression library, we typically load up to 16,500 cells on the 10× Chromium Chip, aiming to profile up to 10,000 cells to minimize cell doublet rate. Please refer to the 10× Genomics Chromium Single Cell 5′ or 3′ Expression protocols for more details. After library preparation and next-generation sequencing, the resulting outputs will be FASTQ sequencing files.

## Quantification and statistical analysis

### Processing of scRNA-seq data occurs in two phases

1. In the initial processing step, we run Cell Ranger (10× Genomics) to align the sequencing reads (FASTQ files) to the human GRCh38 reference genome. This step also generates the cells-by-genes count matrix, which represents the unnormalized expression value (raw counts) of each gene for each profiled cell barcode. The counts matrix can be analyzed in a variety of scRNA-seq software packages, including Seurat,^3^ Monocle3,^7^ Scanpy,^8^ and many others. We conduct most analyses using the Seurat R package,^3^ but regardless of the specific package used, several important steps have become standard practice for secondary processing.
2. The second phase includes doublet filtering, quality control, normalization, batch integration, dimensionality reduction, clustering, and marker identification. Doublets (two or more cells that were captured as a “single” cell during experimental processing) are common and can be identified and filtered using methods such as scDblFinder.^5^ We then calculate a number of metrics for quality control on a per-cell basis, including the number of unique genes detected and the percentages of reads mapping to mitochondrial or ribosomal genes. Low-quality cells (i.e., those with extreme values) are thus identified for removal.
  Batch-specific technical factors can create artifacts that obscure biological signal. Thus batch-correction – also called data integration – is critical. We apply the Harmony method,^4^ although many suitable methods exist. Following integration, we can apply unsupervised clustering (e.g., Louvain or Leiden community detection algorithms) to identify populations of cells with similar transcriptomes. Dimensionality reduction (e.g., uniform manifold approximation projection, or UMAP) enables visualization of the dataset in 2-dimensional space. Finally, differential expression testing (using methods such as MAST^6^) can identify the markers for each cell population. At this point the basic processing is complete, but the true work of analyzing the data – such as the discovery of novel cell types or comparison of disease or treatment conditions begins.

## Limitations

Single-cell RNA-sequencing offers insight into the heterogeneity of skin diseases to identify individual cell-level perturbations within disease states. Researchers should first decide whether a single-cell level of information is necessary for their objectives, or whether traditional bulk RNA-sequencing, where averaged gene expression across a sample is obtained, would suffice. Single-cell RNA-sequencing is much more costly and given the low input for single-cell RNA-sequencing, there can be higher uncertainty when identifying transcriptional states and decreased gene coverage when compared to traditional bulk RNA-sequencing.^9^ This manifests in gene expression dropout, low capture efficiency, and technically noisy data.^10^ If researchers are interested in spatial localization of their transcriptional data, scRNA-seq does not preserve this data and spatial transcriptomics would be the preferred route. Lastly, one must have access to the proprietary 10× Genomics Chromium controller to load samples on to.

There are multiple approaches to isolate cutaneous immune cells. The protocol detailed here is highly optimized for cutaneous immune cells, and if non-immune cell populations are desired, e.g., keratinocytes, this protocol would need to be modified or other approaches utilized. For example, to also capture keratinocytes one would need to separate the epidermis to subject it to harsher trypsin digestion or alternatively, use methods such as the Miltenyi Biotec Whole Skin Dissociation Kit, which combines mechanical dissociation with a proprietary 3 enzyme digestion. Our protocol utilizes enzymatic digestion and 37°C incubation for skin digestion which can lead to enzyme and heat shock associated stress transcriptional responses. However, given the difficulties in dissociating this barrier tissue, most commonly used skin dissociation protocols utilize these steps. One approach to compensate for this is to computationally adjust scRNA-seq results for known tissue digestion stress response genes.^11^^,^^12^

## Troubleshooting

### Problem 1

Low cell numbers.

### Potential solution

This could be due to multiple variables and researchers must identify the issue systematically. If the initial cell count is low in step 17, we note that suboptimal skin biopsy mincing may be an issue. We obtain the best quality results with high-quality scissors for mincing, e.g., Hardened Fine Scissors (Fine Science Tools, FST 14090-11) from the company Fine Science Tools. Additional issues could be faulty centrifugation, discarding excess cells in the supernatant, leaving residual cells and media when transferring between tubes or wells, or suboptimal flow cytometry settings. We noted decreased cell recovery from aspirating supernatant and found that decanting by pouring minimizes cell loss. When decanting after centrifugation, ensure you are not disturbing the cell pellet before removing the supernatant. See Methods video S3 for detail on removing the supernatant. When setting the flow cytometer, it can be helpful to utilize a test sort (if this is an available option) to ensure the sort is calibrated successfully.

### Problem 2

Low cell viability.

### Potential solution

Ensure that you are working quickly, keeping all reagents, tools, and centrifuges at 4°C, using wide-bore pipette tips, pipetting slowly and gently, and minimizing the cell handling you must do.

If the low cell viability occurs before flow sorting, ensure that you are not allowing your digestion to surpass 18 h, and the sample should always be in media unless removing subcutaneous fat. If the low cell viability is after sorting, it is possibly an issue with sorting. First, bring an unstained sample to set your viability gate. Second, ensure your viability dye has not expired, and if you are using DAPI, create a new aliquot of your working solution. Lastly, perform a viability dye titration, as your sample may require a higher concentration of viability dye. Flow sort the cells from the titration and perform a cell count after to ensure you are collecting single, live cells.

### Problem 3

Clogging during flow sorting.

### Potential solution

Ensure that you are filtering your cells through a 100 μm strainer, as mentioned in step 12, and then again through a filtered FACS tube just before sorting. If it clogs again, the sample may be at too high of a concentration, which will also result in a low cell sorting efficiency and lead to cell loss. Increase the sample volume and consider filtering again through a filtered FACS tube.

## Resource availability

### Lead contact

Further information and requests for resources and reagents should be directed to and will be fulfilled by the lead contact, Jeffrey B. Cheng (Jeffrey.cheng@ucsf.edu).

### Materials availability

This study did not generate new unique reagents.

### Data and code availability

No datasets were generated for this protocol. The scRNA-seq data from Cook et al.^1^ and from Liu et al.^2^ is submitted to the European Genome-Phenome Archive (EGA) under accession numbers EGAS00001006716 and EGAS00001005271, respectively. For Cook et al.,^1^ processed Seurat objects are at https://zenodo.org/record/7242347#.Y7Ggn3bMJMy and analysis scripts are available at https://github.com/cpcook1/TTP. For Liu et al.,^2^ processed Seurat objects are available at https://zenodo.org/record/6471748#.Y3-EhUnMJD9 and analysis scripts are available at https://github.com/Yale73/scRNA-seq-for-diverse-human-rashes.
